# The micro revolution: effect of Bite-Sized Teaching (BST) on learner engagement and learning in postgraduate medical education

**DOI:** 10.1186/s12909-021-02496-z

**Published:** 2021-01-21

**Authors:** Kimberly D. Manning, Jennifer O. Spicer, Lucas Golub, Mikhail Akbashev, Robin Klein

**Affiliations:** 1 Department of Medicine, Division of General Internal Medicine, Emory University School of Medicine, 49 Jesse Hill Jr Dr., Atlanta, GA 30303 USA; 2 Department of Medicine, Division of Infectious Diseases, Emory University School of Medicine, Atlanta, GA USA; 3 CareMore Health, Atlanta, GA USA

**Keywords:** Resident education, Didactics, Instructional strategy, Cognitive load theory, Peer assisted learning, Medical education

## Abstract

**Context:**

Bite-sized learning is an instructional method that utilizes brief, focused learning units. This approach may be beneficial in medical education given demands on learner time and cognitive load. This study aims to assess the impact of this approach on knowledge acquisition and learner attitudes in postgraduate medical education.

**Methods:**

An instructional method, termed Bite-Sized Teaching (BST), was implemented within the curriculum at a US Internal Medicine postgraduate training program. In BST, content is distilled into manageable units focused on relevant schemas and delivered via brief peer teaching. A two-fold assessment of BST was performed that included cross sectional survey to assess learner attitudes and experiences and a controlled study to assess knowledge acquisition with BST and case-based teaching control.

**Results:**

One hundred and six of 171 residents (62% response rate) completed the survey. Most residents (79.8%) reported BST was among the best conference types in the curriculum. Important components of BST cited by residents include the distilled content, multiple short talk format and peer teaching. Residents report incorporating what they learned via BST into their teaching (76.1%) and clinical practice (74.1%). Resident who had participated as speaker were significantly more likely to report incorporating learning from BST into their teaching (87.2% vs 63.0%, *p* < 0.01, Cramer’s V effect size = 0.37) and clinical practice (89.7% vs 65.3%, p = 0.02, Cramer’s V effect size 0.28).

Fifty-one residents participated in the knowledge assessment. Residents taught via BST scored significantly higher on immediate post-test compared to case-based teaching (score [SE] 62.5% [1.9] vs 55.2% [2.4], p = 0.03, Hedges g effect size 0.66). While both groups improved over pretest, there was no significant difference in scores between BST and case-based teaching at two (score [SE] 57.1 [2.1] vs 54.8 [2.5], p = 0.54) and six weeks (score [SD] 55.9 [2.1] vs 53.0 [2.9], p = 0.43).

**Conclusions:**

Teaching via brief, focused learning units delivered by peers is well received by learners and appears to have a significantly greater impact on immediate knowledge recall than case-based teaching. Further study on long term knowledge retention and behaviors is needed. Bite-Sized Teaching may be a promising instructional approach in medical education.

**Supplementary Information:**

The online version contains supplementary material available at 10.1186/s12909-021-02496-z.

## Introduction

Health professions education programs are charged with effectively teaching extensive and complex content. The need to acquire a large amount of knowledge is a key stressor for learners in medical training [1]. Educational programs need to consider a variety of instructional methods to meet this challenge [2].

When considering instructional strategies, the unique context of the modern learner must be considered [3, 4]. Learners must contend with multiple competing demands on their time and mental bandwidth and content is vast and complex. Cognitive load theory offers a framework to contend with these factors by positing that individuals have a finite working memory to input, process and retain information [5–7]. Working memory is divided between attending to the complexity of information to be learned (intrinsic load), the demands imposed by distractions not critically relevant to what you are learning (extrinsic load) and effort needed to build schemas for storing information as memory (germane load) [6, 7]. Instructional strategies that manage cognitive load including how content is presented and received have the potential to optimize translation of content into knowledge retention [5–7].

One emerging strategy, often referred to as bite-sized or micro learning, utilizes brief, focused learning units. Often used interchangeably, bite-sized learning employs brief learning units built around a specific objective while microlearning delivers bite-sized learning via educational technologies such as mobile applications and online modules [8, 9]. Bite-sized learning aims to facilitate learning by targeting extraneous load to facilitate working memory [5].

Bite-sized learning may be well suited to health professions education, including professional development and interprofessional education [9–11]. Potential advantages of bite-size learning in health professions education include its ability to contend with cognitive load and ability to use this approach in concert other instructional strategies [5]. Early reports suggest this approach is feasible and viewed positively by learners in postgraduate and undergraduate medical education [12, 13]. To date, literature is largely limited to descriptive report with limited exposure and outcomes reported [9–13]. Evidence of the impact of bite-sized learning on learners and learning in medical education is lacking.

To help address this, we implemented an instructional approach termed Bite-Sized Teaching (BST) that utilizes bite-sized learning principles within an Internal Medicine (IM) residency curriculum. This paper details our instructional approach and an assessment of its impact on knowledge acquisition and learner attitudes. In this work, we use the terms bite-sized learning to refer to the principle of using focused, brief learning units, Bite-Sized Teaching or BST to refer to our approach described here, and didactics to refer to the formal, structured instruction in which BST was implemented.

### Bite-sized teaching

Bite-Sized Teaching (BST) is an instructional approach that incorporates bite-sized learning principles and peer teaching. BST utilizes multiple, focused talks given by learners and delivered within the resident curriculum. The result is replacing a 40 to 50- min teaching session with 4 to 5 brief (8 min) teaching micro-sessions created and delivered by learners. Table 1 provides description and rationale for the key components of BST.

**Table 1:** Rationale for Components of the Bite-Sized Teaching (BST) Model

Component	Description	Rationale
Brief focused learning units	Multiple, 8-min microlearning talks threaded thematically replace traditional lecture.	Brief learning units enable learner engagement [5, 6]. ‘Chunking’ or segmenting content in smaller parts manages intrinsic load [5, 6].
Distilled, relevant content	Provide carefully refined and relevant content focused around one specific learning objective. Organize content in knowledge schemas for learners.	Focus on teaching knowledge schemas for learners to enhance germane load and enable storage in long-term memory [5, 6]. ‘Weeding out’ of non-essential content reduces extraneous cognitive overload and focuses attention on content [14].
Refined presentation & delivery	Prioritize use of select purposeful visual aids and attention to delivery.	Use of media to reduce extraneous load. Includes eliminating redundancy, signaling to focus attention, integration of images and modality effect of using complimentary visual and auditory channels enable learning [14–16].
Peer Teaching	Resident speakers create and deliver talk for audience of peers.	Active learning enables deeper understanding of content. Peer teaching enables consideration of peers’ learning needs, matching content with expertise, and promotes learner engagement to enhance germane load [17, 18].

Using bite-sized learning principles, complex content is broken down and distilled into discrete, manageable units focused on relevant knowledge schemas. Rather than reviewing all content relevant to the topic, a BST talk focuses on a single construct and provides a schema for that construct for learners organize and understand related facts and ideas [6].

**Fig. 1:**
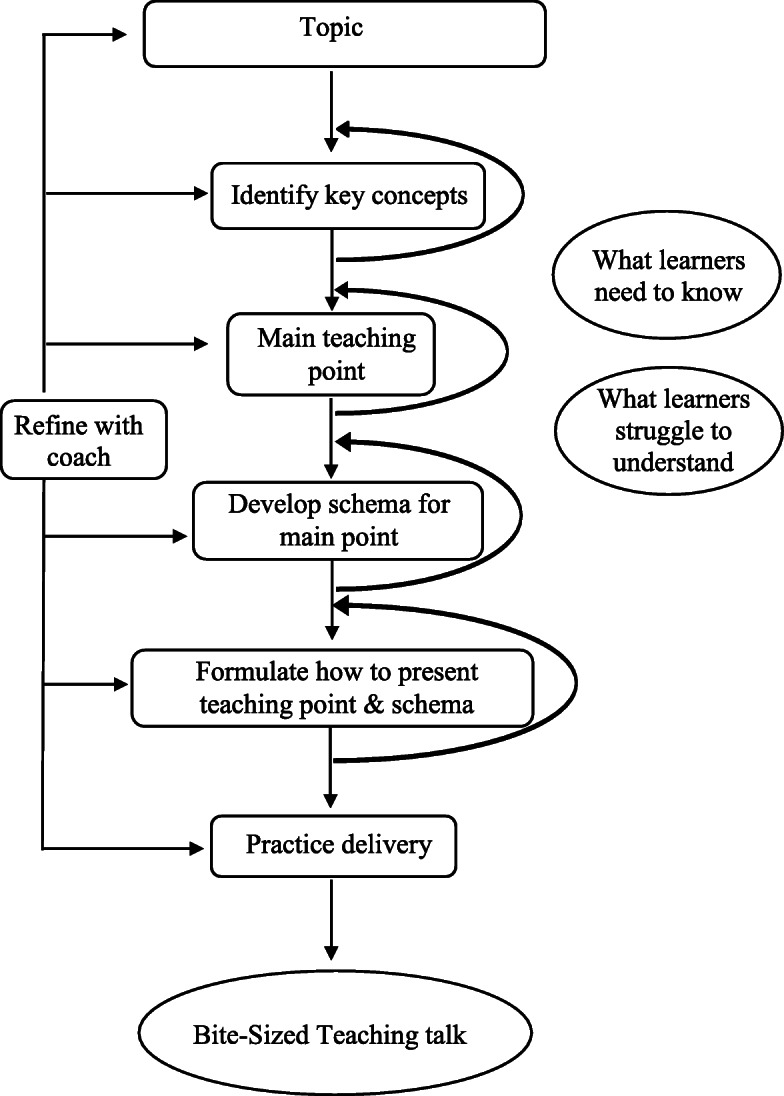
Process of Preparing a Bite-Sized Teaching Talk

Creating a BST talk involves a process of deconstruction and rebuilding. Figure 1 depicts the process of creating a BST talk. Each BST talk is focused around a one main learning objective for those attending the session. Speakers start with a broad area of interest and then deconstruct this into its constituent parts. Speakers continue this process until they have deconstructed down into a single conceptual teaching point that they want learners to take away from the session. Speakers then build a framework or schema to support and explain their teaching point. Talks are crafted around this knowledge schema; Content directly and critically related to this schema is included and non-essential content is removed [14].

The process of distillation and building is iterative and often requires multiple passes. Resident speakers work closely with a faculty coach with content and teaching expertise to refine content. Throughout this process, speakers consider learner expertise, gaps in learner understanding, and concepts most relevant to learners [5]. A crucial point, the aim of a bite-sized teaching talk is not to condense an hour of material into 8 min, but rather to distill content to what is most essential to the learner and their learning.

Cognitive load principles to enable learning are used to guide the structure and presentation of BST talks, including audiovisual materials and delivery. Thoughtfully constructed verbal and pictorial representations enable learners to process and retain knowledge schema [14–16]. This includes optimizing the use of visual and auditory channels to convey information, eliminating unnecessary images and text, and signaling to emphasize main points [14–16].

Each BST conference has a broad theme to thread short talks together and address core curricula needs. Participation is voluntary and speakers are recruited from those on less time intensive rotations. We send anticipatory messages with theme, topics, speaker selfies and BST-specific hashtags (#everybodylearning #everybodyteaching) via e-mail and social media. Talks are delivered in-person or streamed live online at a time reserved for resident education.

We utilize BST as an instructional method within the IM resident didactic curriculum at Emory University. This curriculum provides formal teaching for residents in the core components of the field and uses a variety of instructional methods including lecture, peer teaching, and case-based discussion. The IM curriculum is divided into ambulatory, inpatient and critical care teaching conferences and these are delivered during time reserved for resident education. We added BST to our curriculum in 2014 and currently host 1 to 3 BST sessions monthly in the inpatient curriculum. Since its introduction, we have hosted over 50 BST conferences and residents have created and delivered more than 200 unique Bite Size Teaching talks within the IM residency curriculum.

## Methods

**Fig. 2:**
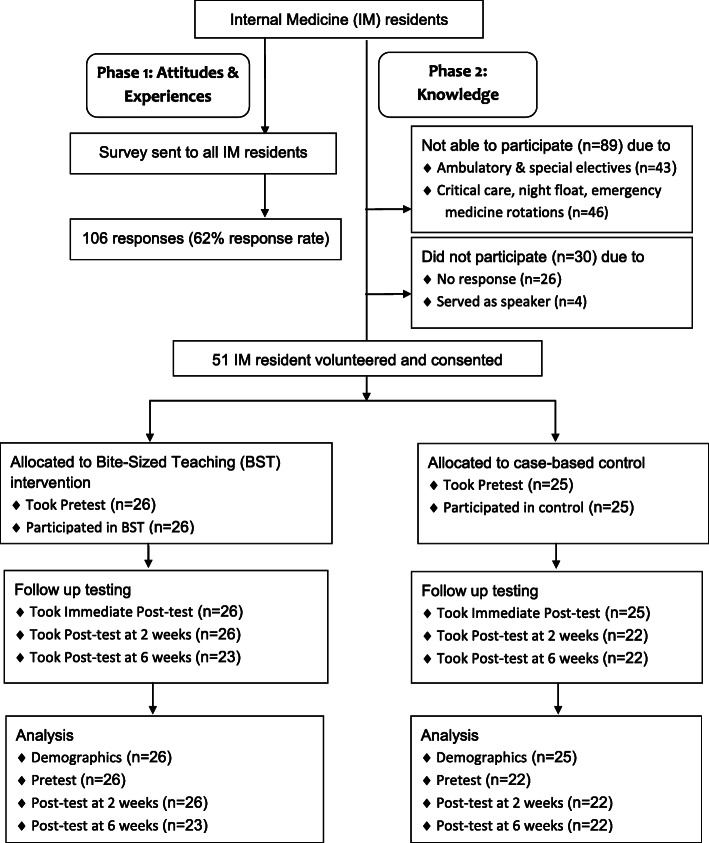
Approach to Assessment of Bite-Sized Teaching in Internal Medicine resident curriculum

This study aims to assess the impact of BST on learner attitudes, experiences and knowledge acquisition in postgraduate medical education. We performed a two-phase assessment Bite-Sized Teaching (BST). See Fig. 2. The first phase assessed learner attitudes toward and experiences with BST and its components. The second phase assessed learner knowledge acquisition with BST. Participants were residents from the IM training program at an academic institution in the US.

### Phase 1: attitudes and experiences

To assess resident attitudes and experience with BST as part of the IM didactic curriculum, we designed and performed a cross-sectional survey of all IM residents in our program. To do this, we conducted a focus group of residents who had participated in BST as speaker and learner during their training. We then created a 14-question item survey that included closed and open ended questions focused on learner attitudes towards BST and its components and experiences with BST during training. Survey was piloted and refined with IM faculty.

Survey was administered to all 170 residents in the IM residency training program via an online survey tool in January 2018.

### Phase 2: knowledge

To assess impact on learning, we conducted a controlled study comparing knowledge acquisition with BST comparted to an interactive case-based teaching session as a control. We reviewed the existing IM resident core curriculum and identified transfusion medicine as a clinical content area that residents would need to know but lacked dedicated instruction in the current inpatient IM curriculum. We identified learning objectives and key topics deemed important for IM postgraduate trainees based the literature [19, 20]. See Additional file 1: Table S1. These were used to create BST and case-based teaching sessions to ensure consistency between approaches.

We recruited participants via email from IM residents on inpatient rotations in April and May of 2019. Participants attended either the BST or case-based teaching sessions and participants were assigned to BST or control groups based on the hospital site at which they were rotating. This was done to minimize confounding exposure to the alternate teaching strategy. Scheduling of resident rotations is done by program leaders a year in advance.

Participation was voluntary and provided informed consent prior to participation.

Teaching sessions were delivered live, in-person during the time reserved for inpatient IM resident teaching conferences. IM residents and faculty served as speakers for BST and control sessions, respectively. In BST, four IM residents delivered multiple 8-min BST teaching talks under the guidance of one author (KM). For the control, one author (AB) with teaching and content expertise delivered a 50-min interactive, case-based teaching session. This control session used case-based teaching in which cases representing authentic clinical scenarios are presented and analyzed with learners to promote application of knowledge. Key aspects of this include interaction between learners and teacher and linkage of learning to relevant clinical scenarios. Talks were delivered in 4 conference sessions by the same speakers to ensure consistency.

We utilized a 20-item multiple choice test that tests factual and applied knowledge in transfusion medicine created by the Biomedical Excellence for Safe Transfusion Collaborative Transfusion test [20, 21]. This test has been validated with learners in medical education setting and has an expected basic knowledge score of 42% in postgraduate learners [21]. The same test was used for all testing events. One author (RK) administered the tests and speakers (including KM and AB) were blind to test questions and answers. Participants took a pre-test approximately 1 week prior to the teaching session and post-tests immediately, at two weeks, and six weeks following the teaching sessions. With the pre-test, participants completed a brief questionnaire that asked residents to rate their interest in the field and preference for various teaching formats.

### Analysis

We calculated descriptive statistics including mean, standard deviation, and distribution. Yates Chi-square corrected for continuity was used to assess associations between groups in survey responses. Cramer’s V was used to calculate effect size with an effect size of 0.1 was considered small, 0.3 was considered moderate and 0.5 was considered large effect [22, 23]. Focus group comments and responses to open-ended survey questions were analyzed using qualitative analysis [24].

For the knowledge assessment, we compared mean scores on pre-test with subsequent post-tests using paired Wilcoxon signed-rank test in both BST and control groups. Mean test scores between BST and control groups were compared using Wilcoxon rank sum test. Bias corrected Hedges g was used to calculate effect size in testing with an effect size of 0.2 was considered small, 0.5 was considered moderate and 0.8 was considered large effect [22, 23]. A *p*-value ≤ 0.05 was used for statistical significance.

Study protocol was approved by the Institutional Review Board at Emory University and methods were carried out in accordance with relevant guidelines and regulations. Participants provided informed consent to participate in study activities.

## Results

### Attitudes and experiences

**Fig. 3:**
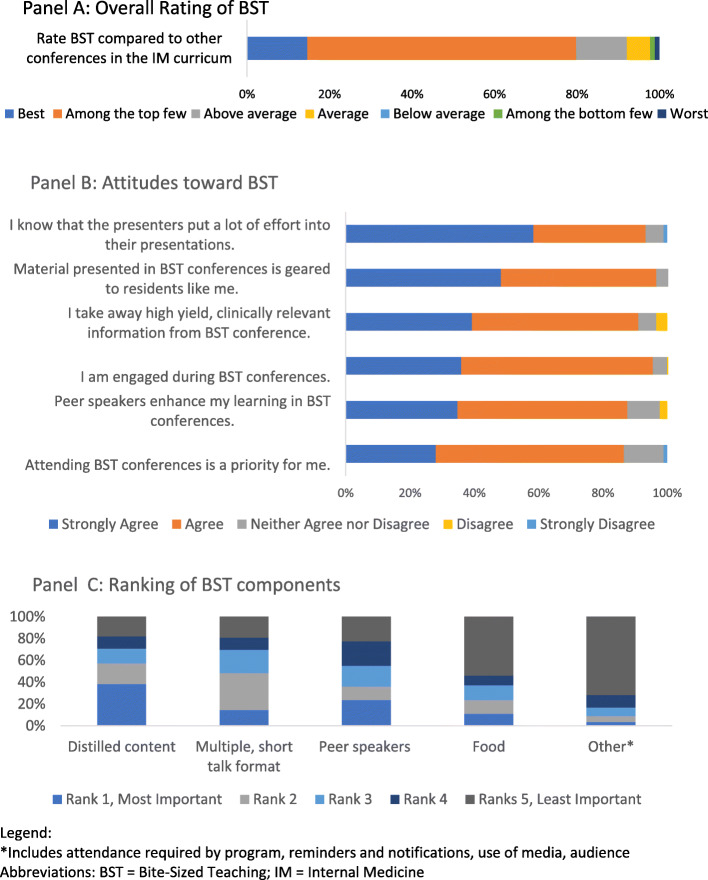
Learner attitudes towards Bite-Sized Teaching and its components via participant survey

One hundred and six of 170 IM residents completed the BST survey (62% response rate). Figure 3 depicts survey findings. Majority of residents (79.8%) reported BST was one of or the best conference type used in the IM curriculum. Majority of residents reported being engaged with BST, that BST content was highly relevant and geared towards residents. The most important components of BST as ranked residents include the distilled content, multiple short talk format and peer teaching.

Forty residents reported having experience creating and delivering a BST talk during their training. The majority of residents who had participated as BST speaker reported that the BST speaker experience helped to develop presentation skills (90%) and they apply the teaching skills learned from BST in their teaching (87.5%). Residents who had participated as BST speaker report the speaker experience affected how they teach and the frequency with which they teach (87.2 and 51.3%, respectively).

Residents report incorporating what they learned via BST into their teaching (76.1%) and clinical practice (74.1%). Resident who had participated as speaker were significantly more likely to report incorporating learning from BST into their teaching (87.2% vs 63.0%, *p* < 0.01, Cramer’s V effect size = 0.37) and clinical practice (89.7% vs 65.3%, p = 0.02, Cramer’s V effect size 0.28).

Seventy-five participants provided narrative comments concerning their impression of BST. Thematic analysis of comments identified key themes related to learners’ attitudes toward BST and its components and experience with BST. Themes and representative comments are detailed in Table 2. Residents recognized and valued key components of BST including the brief talk format, distilled and relevant content, how BST is presented, and being taught by peers. Residents found BST to be an overall valuable approach to didactic conference and noted the individual experience as BST speaker was noteworthy.

**Table 2:** Resident attitudes and experience with Bite-Sized Teaching from qualitative data

Themes		Representative Comments
Components of Bite-Sized Teaching	Multiple, brief talks	PGY2: *“It’s what medical education should be. Nobody in medical school can pay attention for 4 h straight … In residency, our attention is even more divided. Topics should go no longer than 30 min at a time. So the 8-min lecture is ideal.”* PGY2: “W*hen it’s only 8 min, people will pay attention for the whole 8 min. It really forces you to be on your game, make everything count.”* PGY3: “*As someone who gets his phone out frequently during conference, the frequency that I do that during BST is much lower. Part of that is that the topics are interesting, part of it is the voice is changing every 8 min. I get itchy watching people stand up and talk for an hour. And they’ve lost me at 12:07. Like, I have completed the Internet by 1 PM.”*
	Content	PGY1: “*Bite-size information is easy to digest, sticks well during a busy day.”* PGY2: “*High yield information, relevant and not too much detail, makes it more manageable.”* PGY3*:*” *I just want to know what I need to know. Like when we have 45-min presentations and like half of them are this study, I will tune out and get lost. BST is great for getting rid of that. Distilling out.”*
	Presentation & delivery	PGY3*: “Succinctness of the presentation is the most important thing. All that needed be ingested was a sentence as opposed to a paragraph or literally an entire slide of words. Use (visual aids) to amplify key points. Because if there’s not a lot on there, the audience doesn’t have to digest very much.”* PGY3*:”* D*elivery is key. It’s delivered in a way that no other talks are delivered. It’s not formal and not a lot of studies, graphs and charts. It’s like you’re teaching somebody”*
	Peer Teaching	PGY2: “*If I know that it’s something that can be understood by someone at my level, I feel like that’s knowledge that I should have ... As opposed to someone who’s been studying something for longer than I have been alive, and they drop some knowledge, I’m not sure I need to know that.”* *PGY2: “Excellent way to learn and teach (And learn how to teach!) … and I love that we are motivated by the fact that we present to our peers and they do depend on the knowledge we share to be applicable and accurate. Overall, they are usually very well done- and that expectation remains for most people when they attend.”* *PGY3: “You buy into it because it’s your peer.”*
Experience	Speaker experience	PGY3: “*it helped me sharpen my public speaking, identify relevant aspects of clinical teaching and made me an expert in the subject I was teaching.”* PGY3: “*Made me learn the content because to bring something (down) to what you absolutely need to know, you need to know it well, to really respect what is the most vital thing.”*
	Overall experience	PGY3: *“BST mode is an excellent format, with a built-in structure that guides the speaker to adopt more engaging styles and better identify the learning points relevant to the listener, presented in a format well-suited to integrating this information. It is a highlight of our conferences at this institution.”*

*Abbreviations*: *BST* Bite-sized teaching, *PGY1* post-graduate year 1, *PGY2* post-graduate year 2, *PGY3* post-graduate year 3

### Knowledge

To assess knowledge acquisition, 51IM residents participated in testing and teaching events. Table 3 details the demographics of BST and control groups participating in the knowledge assessment. There were no significant differences between BST and control groups in participant number, post graduate year, or preference for BST or case-based teaching. Given the topic of transfusion medicine, we assessed for interest in hematology and oncology career between groups and found no difference.

**Table 3:** Demographics of Participants in Assessment of Bite-Sized Teaching on Knowledge

	Bite-Sized Teaching (BST)	Interactive Case-based Teaching	*P* value^a^
Participants completing pretest, N	26	25	0.50
Participants completing posttest1, N	26	25	
Participants completing posttest2, N	26	22	
Participants completing posttest3, N	23	22	
Participants			
PGY 1, *n* (%)	11 (42%)	9 (36%)	0.87
PGY 2, *n* (%)	9 (35%)	8 (32%)	
PGY 3, *n* (%)	6 (23%)	8 (32%)	
Interested in hematology oncology career, *n* (%)	4 (15%)	4 (16%)	0.94
Preferred didactic format			
Case-based conference, *n* (%)	20 (77%)	21 (84%)	0.47
Lecture, *n* (%)	6 (23%)	5 (20%)	0.64
Bite-Sized Teaching, *n* (%)	20 (77%)	18 (72%)	0.94

*Abbreviations*: *BST* Bite-sized teaching, *PGY1* post-graduate year 1, *PGY2* post-graduate year 2, *PGY3* post-graduate year 3

^a^ *P* value comparing scores of BST intervention to interactive case-based control

**Fig. 4:**
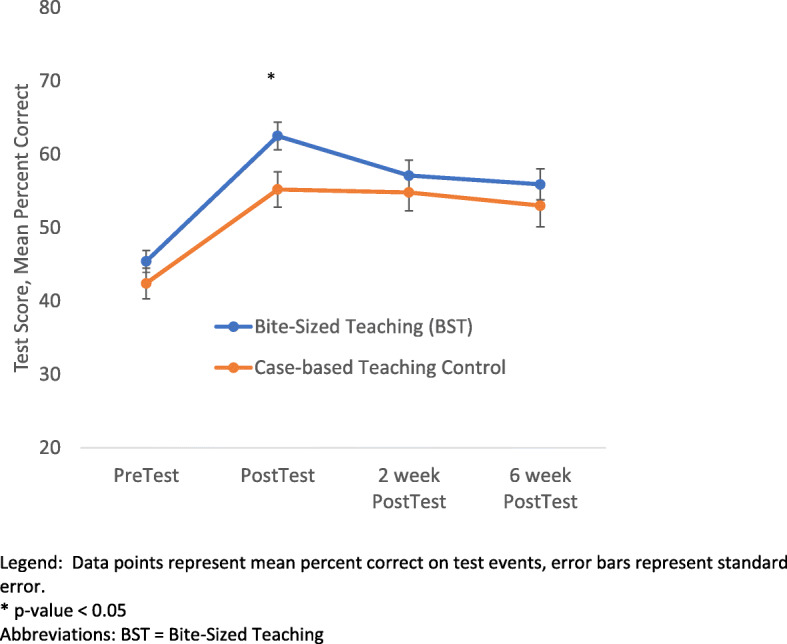
Learning Outcomes with Bite-Sized Teaching compared to Case-based Teaching Control via Pre and Post testing

Figure 4 depicts BST and control group scores on testing events. Both BST and control groups scored higher on all post-test events than pretest score in paired analysis. See Additional file 2: Table S2. BST and control groups scored similarly in pretest (pretest score [SE] 45.4% [1.5] vs 42.3% [2.1], p = 0.33). In the immediate post-test, the BST group scored significantly higher than the control group (score [SE] 62.5% [1.9] vs 55.2% [2.4], Hedges g effect size= 0.66, p = 0.03). There was no significant difference in post test scores between BST and control groups at two (score [SE] 57.1 [2.1] vs 54.8 [2.5], p = 0.54) and six weeks (score [SD] 55.9 [2.1] vs 53.0 [2.9], p = 0.43).

## Discussion

When implemented within an IM resident curriculum, we found a positive impact on learner attitudes, experiences and knowledge acquisition with the BST approach. This suggests that bite-sized learning as implemented in our intervention may be a useful teaching approach in GME.

When implemented routinely as a teaching strategy in didactics, the BST model was perceived positively by learners. Notably, residents reported being engaged with conferences using this approach. Studies have demonstrated resident preference for shorter sessions, resident speakers, and focus on high-yield pearls [25, 26]. Our findings are consistent with prior work that noted learner satisfaction with BST, although exposure was limited [12]. When used in the context of professional development, viewings of online modules increased when brief focused sessions, were utilized compared to longer sessions [9].

We found the majority of residents reported changing their teaching and clinical practices in response to what they learned via BST. For teaching activities in particular, content packaged into brief focused units may make BST content more accessible to residents in their own teaching practices. Evidence of a link between bite-sized learning and impact on behaviors is limited. Study using brief simulation exercises reported improvements in self-reported confidence in managing certain emergency situations [10]. While promising, further study of the impact on behaviors is warranted.

Although not a core component of bite-sized learning, peer teaching is a key component of BST as described here. While faculty and near peer speakers such as medical students and fellows have also participated in BST conferences as speaker, most BST conferences employ resident speakers. Peer teaching was one of the components of BST ranked by learners as most important behind relevant, distilled content and multiple short talk format. The use of peer teaching in BST was intentional as this provided speakers with an active learning experience [27]. Meta-analysis of peer teaching in medical education found comparable impact on knowledge and skills outcomes with peer compared to faculty teaching but noted peer teaching provided benefits in terms of developing teaching skills [28].

Our findings suggest that the BST speaker role may be a particularly impactful experience. We found that experience as a speaker in BST was beneficial in honing teaching skills and the speaker experience affected how and how often they teach. Narrative comments support this as we found resident speakers cultivate content mastery and teaching skills. These skills may then translate to their teaching and clinical practices as residents who serve as speaker reported changes in teaching and clinical practice behaviors at higher rates than non-speakers.

Active learning approaches in inpatient resident didactics poses challenges due to competing demands on resident time and cognitive bandwidth [29, 30]. We found the BST approach to be a feasible means incorporating components of active learning within the resident curriculum as it shifts time intensive preparatory activities to those with the bandwidth to successfully manage this task and distributes the load of peer teaching across residents.

Bite-sized learning and peer teaching may have complimentary effects on engagement and learning [5]. Peer assisted learning approaches such as peer teaching are thought to enable learning via cognitive and social congruence [17, 18]. Cognitive congruence posits that peers have a unique understanding of the learning needs, deficiencies and challenges of their peers [17, 18]. While faculty have content expertise, residents are experts in what residents know and what they struggle to understand. Social congruence involves the interpersonal aspect of peer learning and posits that social bonds between peers can drive learning [17, 18]. Narrative comments suggest that residents appreciate schemas framed through the lens of a peer and that social bonds prompt them to ‘show up’ for each other both as learner and speaker.

We found a significant, moderate size impact on immediate knowledge scores of IM residents with BST teaching compared to case-based discussion using validated assessment tool. Both BST and case-based teaching had significant improvements in scores in immediate and delayed post-testing events as compared to pretest. There was no significant difference in delayed post-test scores with BST over case-based teaching, although study power may play a role. Findings suggest that BST may have a positive impact on knowledge acquisition. When implemented in the form of high-impact, 10-min talks delivered in a clinical workplace setting, Bartam et al. found improvements in perceived knowledge of participants with bite-sized learning [11].

Impact on knowledge acquisition with BST was noted when used as a teaching approach within an IM inpatient curriculum. Studies have shown a lackluster impact on learning with traditional didactic approaches [31–33]. Teaching strategies of benefit in formal teaching conferences, especially inpatient didactics, are a particular need in postgraduate medical education [29, 30]. Findings suggest bite-sized learning may be particularly useful in settings with high cognitive load, such as teaching conferences geared towards residents on inpatient rotations. When implemented in the context of didactic conference, BST may serve to redistribute cognitive load to enable working memory. Narrative comments support this notion as the short talk format enabled engagement and participants appreciated distilled content.

Limitations of our work reliance on resident self-reported impact on attitudes and behaviors and multiple choice test to assess learning. While we used a validated test, this does not adequately capture impact on higher level learning processes [34]. It is possible that score differences relate to differences between groups or the impact of repeated testing effects rather than learning through BST, although the patterns in scores and demographic variables between groups does not suggest this. While promising, resident-reported changes in teaching and clinical practice behaviors warrants further study. Sample size of knowledge assessment may affect ability to discern impact on long term retention and larger scale study is needed. Volunteer response and social desirability bias may influence survey findings. The single institution, single specialty population may limit generalizability and more study is needed of BST in other learner settings.

Bite-sized learning in medical education is ripe for study given its ability to contend with context of the modern learner in health professions education. Advantages of this approach include the ability to adapt quickly to shifts in context, content and learner needs [8]. To date, the BST model has been implemented in the postgraduate and undergraduate curricula at our institution [12, 13] and other institutions including the Massachusetts General Hospital, Boston University, and Medical College of Wisconsin. (Kerri Palamara McGrath, MD, email communication, October 2020; Gopal Yadavalli, MD, e-mail communication, February 2018; Marty Muniz, MD, and Pat Foy, MD, e-mail communication, June 2016).

## Conclusion

Bite-Sized Teaching is an instructional strategy that employs peer teaching and bite-sized learning principles to manage cognitive load. When implemented in postgraduate IM training curriculum, BST has a positive impact on resident attitudes and immediate knowledge recall compared to case-based teaching. Bite-sized learning as implemented in BST may be a promising instructional strategy in postgraduate medical education.

## Supplementary Information

**Additional file 1: Table S1.** Transfusion Medicine Topics and Learning Objectives used for intervention and control teaching sessions.

**Additional file 2: Table S2.** Impact of Bite-Sized Teaching and Case-based teaching on pre and post test scores.

## Data Availability

Transfusion test available from Biomedical Excellence for Safer Transfusion (BEST) Collaborative. Data generated and analyzed for the current study is available from the corresponding author on reasonable request.

## References

[1] Radcliffe C, Lester H (2003). Perceived stress during undergraduate medical training: a qualitative study. Med Educ.

[2] Pluta WJ, Richards BF, Mutnick A (2013). PBL And beyond: trends in collaborative learning. Teach Learn Med.

[3] O’Malley PG, Khandekar JD, Phillips RA (2005). Residency training in the modern era: the pipe dream of less time to learn more, care better, and be more professional. Arch Intern Med.

[4] Lujan HL, DiCarlo SE (2006). Too much teaching, not enough learning: what is the solution?. Adv Physiol Educ.

[5] Young JQ, Van Merrienboer J, Durning S, Ten Cate O (2014). Cognitive load theory: implications for medical education: AMEE guide no. 86. Med Teach.

[6] Mayer RE (2010). Applying the science of learning to medical education. Med Educ.

[7] Van Merriënboer JJ, Sweller J (2010). Cognitive load theory in health professional education: design principles and strategies. Med Educ.

[8] Hug T. Didactics of microlearning. Waxmann Verlag; 2007.

[9] Stahl SM, Davis RL, Kim DH, Lowe NG, Carlson RE, Fountain K, Grady MM (2010). Play it again: the master psychopharmacology program as an example of interval learning in bite-sized portions. CNS Spectr.

[10] Sunley R, Moloney K, Parker J (2017). 55 ‘Mini Sim’ – innovative bite sized simulation teaching in a busy children’s emergency department. Emerg Med J.

[11] Bartram R, Dias R, Thompson S, Das A, Nirodi P, El-Sayeh H, Narayna U (2017). Bite sized teaching: delivering knowledge of physical health issues in mental health settings. Br J Mental Health Nurs.

[12] Schwartz AC, Cotes RO, Kim J, Ward MC, Manning KD (2019). Bite-sized teaching: engaging the modern learner in psychiatry. Acad Psychiatry.

[13] DeSimone AK, Haydek JP, Sudduth CL, LaBarbera V, Desai Y, Reinertsen E, Manning KD (2017). Encouraging student interest in teaching through a medical student teaching competition. Acad Med.

[14] Chandler P, Sweller J (1991). Cognitive load theory and the format of instruction. Cogn Instr.

[15] Mayer RE, Moreno R (2003). Nine ways to reduce cognitive load in multimedia learning. Educ Psychol.

[16] Sweller J, van Merrienboer JJG, Paas FGWC (1998). Cognitive architecture and instructional design. Educ Psychol Rev.

[17] Ten Cate O, Durning S (2007). Dimensions and psychology of peer teaching in medical education. Med Teach..

[18] Lockspeiser TM, O'Sullivan P, Teherani A, Muller J (2008). Understanding the experience of being taught by peers: the value of social and cognitive congruence. Adv Health Sci Educ Theory Pract.

[19] Lin Y, Cserti-Gazdewich C, Callum J (2015). University of Toronto Transfusion Camp Organizing Committee. Evaluation of “Transfusion Camp,” a postgraduate transfusion medicine education program using the BEST-TEST knowledge assessment tool. Transfusion..

[20] Haspel RL, Lin Y, Mallick R, Tinmouth A, Cid J, Eichler H, Lozano M, van de Watering L, Fisher PB, Ali A, Parks E (2015). Internal medicine resident knowledge of transfusion medicine: results from the BEST-TEST international education needs assessment. Transfusion..

[21] Haspel RL, Lin Y, Fisher P, Ali A, Parks E (2014). And biomedical excellence for safer transfusion (BEST) collaborative. Development of a validated exam to assess physician transfusion medicine knowledge. Transfusion..

[22] Cohen J (1988). Statistical power analysis for the behavioral sciences.

[23] Sun S, Pan W, Wang LL (2010). A comprehensive review of effect size reporting and interpreting practices in academic journals in education and psychology. J Educ Psychol.

[24] Elo S, Kyngäs H (2008). The qualitative content analysis process. J Adv Nurs.

[25] Sawatsky AP, Zickmund S, Berlacher K, Lesky D, Granieri R (2014). Understanding resident learning preferences within an internal medicine noon conference lecture series: a qualitative study. J Grad Med Educ.

[26] Fraser T, Sargsyan Z, Baggett TP, Baggett M (2016). Quantitative study of the characteristics of effective internal medicine noon conference presentations. J Grad Med Educ..

[27] Ten Cate O, Durning S (2007). Peer teaching in medical education: twelve reasons to move from theory to practice. Med Teach.

[28] Rees EL, Quinn PJ, Davies B, Fotheringham V (2016). How does peer teaching compare to faculty teaching? A systematic review and meta-analysis. Med Teach.

[29] Allenbaugh J, Spagnoletti C, Berlacher K (2019). Effects of a flipped classroom curriculum on inpatient cardiology resident education. J Grad Med Educ..

[30] Cooper AZ, Hsieh G, Kiss JE, Huang GC (2017). Flipping Out: Does the Flipped Classroom Learning Model Work for GME?. J Graduate Med Educ.

[31] FitzGerald JD, Wenger NS (2003). Didactic teaching conferences for IM residents: who attends, and is attendance related to medical certifying examination scores?. Acad Med.

[32] Cacamese SM, Eubank KJ, Hebert RS, Wright SM (2004). Conference attendance and performance on the in-training examination in internal medicine. Med Teach.

[33] McDonald FS, Zeger SL, Kolars JC. Factors associated with medical knowledge acquisition during internal medicine residency. J Gen Intern Med 2007;22(7):962–968. doi: 10.1007/s11606-007-0206-4. Epub 2007 Apr 28. 10.1007/s11606-007-0206-4 PMC2219722 17468889

[34] Wyse AE, Viger SG (2011). How item writers understand depth of knowledge. Educ Assess.

