# Supplementary material for: The micro revolution: effect of Bite-Sized Teaching (BST) on learner engagement and learning in postgraduate medical education
Source: BMC Med Educ. 2021 Jan 21;21:69. doi: 10.1186/s12909-021-02496-z (PMC7819162; doi:10.1186/s12909-021-02496-z)
Supplement: Supplementary file 1 — Additional file 1: Table S1. Transfusion Medicine Topics and Learning Objectives used for intervention and control teaching sessions. [file 12909_2021_2496_MOESM1_ESM.docx]

Appendix Table 1: Transfusion Medicine Topics and Learning Objectives for BST and Case-based Teaching

| Transfusion Medicine Topics and Learning Objectives for BST and Case-based Teaching | | |
| --- | --- | --- |
| **Topics in Transfusion Medicine**  **relevant to IM residents^a^** | **Learning Objectives^a^** |  |
| Transfusion of blood products  -RBC transfusion thresholds  -Platelet transfusion thresholds  -Indications for transfusion  -Transfusion in special populations & scenarios  -Transfusion of other blood products  -Assessing response to transfusion  Transfusion reactions  -Types (febrile, hemolytic, serious, etc.)  -Diagnosis  -Management  -Incidence and Risk  -Prevention | Understand RBC transfusion thresholds and when to apply thresholds in clinical practice. |  |
|  | Review platelet transfusion thresholds and how clinical scenarios relate to platelet transfusion thresholds in clinical practice. |  |
|  | Develop an approach to diagnosis and managing common transfusion reactions. |  |
|  | Distinguish between serious transfusion reactions. |  |
| ^a^ Informed by literature^18,19^ | | |
