# Supplementary material for: The micro revolution: effect of Bite-Sized Teaching (BST) on learner engagement and learning in postgraduate medical education
Source: BMC Med Educ. 2021 Jan 21;21:69. doi: 10.1186/s12909-021-02496-z (PMC7819162; doi:10.1186/s12909-021-02496-z)
Supplement: Supplementary file 2 — Additional file 2: Table S2. Impact of Bite-Sized Teaching and Case-based teaching on pre and post test scores. [file 12909_2021_2496_MOESM2_ESM.docx]

Appendix Table 2: Knowledge Pre and Post Testing in BST and Control Groups

|  | Pretest score | Immediate Post-test score | P  value^a^ | 2-week  Post-test score | P value^a^ | 6-week  Post-test score | P  value^a^ |  |
| --- | --- | --- | --- | --- | --- | --- | --- | --- |
| Bite-Sized Teaching group,  Mean (SD) [n] | 45.4% (7.9) [26] | 62.5% (9.8) [26] | <0.01 | 57.1% (10.7) [26] | <0.01 | 55.9%  (10.1) [23] | 0.01 |  |
| Case-based teaching group, Mean (SD) [n] | 42.3% (10.4) [25] | 55.2% (12.2) [25] | 0.01 | 54.8% (11.7) [23] | 0.01 | 53.0%  (13.8) [22] | 0.02 |  |
| ^a^ P value comparing post-test and pretest scores using paired analysis within groups | | | | | | | | |
